# Comparative genomics and DNA methylation analysis of *Pseudomonas aeruginosa* clinical isolate PA3 by single-molecule real-time sequencing reveals new targets for antimicrobials

**DOI:** 10.3389/fcimb.2023.1180194

**Published:** 2023-08-18

**Authors:** Zijiao Li, Xiang Zhou, Danxi Liao, Ruolan Liu, Xia Zhao, Jing Wang, Qiu Zhong, Zhuo Zeng, Yizhi Peng, Yinling Tan, Zichen Yang

**Affiliations:** ^1^Department of Plastic and Cosmetic Surgery, Xinqiao Hospital, The Second Affiliated Hospital, Army Medical University, The Third Military Medical University, Chongqing, China; ^2^Cadet Brigade 4, College of Basic Medicine, Army Medical University, The Third Military Medical University, Chongqing, China; ^3^Department of Microbiology, Army Medical University, The Third Military Medical University, Chongqing, China; ^4^Institute of Burn Research, State Key Laboratory of Trauma, Burns and Combined Injury, Southwest Hospital, The First Affiliated Hospital, Army Medical University, The Third Military Medical University, Chongqing, China

**Keywords:** *Pseudomonas aeruginosa*, SMRT, comparative genome analysis, DNA methylation analysis, epigenetics, antibacterial

## Abstract

**Introduction:**

*Pseudomonas aeruginosa* (*P.aeruginosa*) is an important opportunistic pathogen with broad environmental adaptability and complex drug resistance. Single-molecule real-time (SMRT) sequencing technique has longer read-length sequences, more accuracy, and the ability to identify epigenetic DNA alterations.

**Methods:**

This study applied SMRT technology to sequence a clinical strain *P. aeruginosa* PA3 to obtain its genome sequence and methylation modification information. Genomic, comparative, pan-genomic, and epigenetic analyses of PA3 were conducted.

**Results:**

General genome annotations of PA3 were discovered, as well as information about virulence factors, regulatory proteins (RPs), secreted proteins, type II toxin-antitoxin (TA) pairs, and genomic islands. A genome-wide comparison revealed that PA3 was comparable to other *P. aeruginosa* strains in terms of identity, but varied in areas of horizontal gene transfer (HGT). Phylogenetic analysis showed that PA3 was closely related to *P. aeruginosa* 60503 and *P. aeruginosa* 8380. *P. aeruginosa*'s pan-genome consists of a core genome of roughly 4,300 genes and an accessory genome of at least 5,500 genes. The results of the epigenetic analysis identified one main methylation sites, N6-methyladenosine (m6A) and 1 motif (CATNNNNNNNTCCT/AGGANNNNNNNATG). 16 meaningful methylated sites were picked. Among these, *purH*, *phaZ*, and *lexA* are of great significance playing an important role in the drug resistance and biological environment adaptability of PA3, and the targeting of these genes may benefit further antibacterial studies.

**Disucssion:**

This study provided a detailed visualization and DNA methylation information of the PA3 genome and set a foundation for subsequent research into the molecular mechanism of DNA methyltransferase-controlled *P. aeruginosa* pathogenicity.

## 1 Introduction

*Pseudomonas aeruginosa*, a gram-negative bacterium is one of the top three causes of opportunistic human infections which inherent resistance to antibiotics and disinfectants (Stover et al., 2000). *P. aeruginosa* takes responsibility for many nosocomial infections, including pneumonia, surgical site infection, urinary tract infection, and bacteremia (Reynolds and Kollef, 2021). In addition, *P. aeruginosa* is the most prevalent multidrug-resistant infection in hospitalized patients, especially in ICU or burn wards (Parkins et al., 2018). An improved understanding of the pathogenicity and resistance mechanisms of *P. aeruginosa* has led to the development of novel antimicrobial approaches (Dimopoulos et al., 2020). However, there is still an urgent need in investigating its genomics under the circumstances of the rapid mutation and evolution of *P. aeruginosa*.

Whole-genome sequencing is commonly used to analyze and learn the genomic background of an organism. Third-generation sequencing, often characterized by single-molecule sequencing, with ultra-long read lengths and higher accuracy (Ardui et al., 2018), fundamentally differs from clone-based second-generation sequencing approaches (Gu et al., 2019). The current third-generation sequencing technology, single-molecule real-time (SMRT) sequencing from Pacific Biosciences (PacBio) directly detects DNA methylation without bisulfite conversion (Eid et al., 2009). This allows single-molecule identification of epigenetic modifications at base resolution. And the long read length of SMRT sequencing may allow motif analysis in highly repetitive genomic regions (Flusberg et al., 2010). Thus SMRT sequencing has significant implications for enhancing the understanding of epigenetic and phenotypic diversity (Li et al., 2016; Attar, 2016) through whole-genome sequencing, targeted sequencing, complex population analysis, RNA sequencing, and epigenetic characterization (Nakano et al., 2017).

Restriction-modification system (RM system) in *P. aeruginosa* mainly consists of restriction enzymes and methyltransferases, that protect individuals from foreign DNA (such as bacteriophages) intrusion (Pleška et al., 2016). Generally, the RM system consists of a methyltransferase (MTases) with a target recognition domain (TRD) that can function independently and another restriction endonuclease that can only bind to DNA. The epigenetic control of *P. aeruginosa* is mainly achieved through the DNA MTases (Anton and Roberts, 2021) by transferring methyl groups from donor S-adenosyl-l-methionine (SAM) to specific positions on target bases to form distinct modifications (Gold et al., 1963). This specific location is called the motif, where MTase is capable of modifying a base (typically between 4 and 8 bp long). Recently, bacterial DNA methylation mediated by MTases has been found to play multiple important roles in regulating gene expression, virulence, pathogen-host interactions, and antimicrobial resistance (Cohen et al., 2016). Some highly conserved DNA MTases may serve as promising targets for developing novel antibiotics through epigenetic regulations (Oliveira and G Fang, 2021).

The genome of different *P. aeruginosa* strains vary during evolution when methylation brings even greater epigenetic adaptations. The genome-wide DNA methylation pattern of a classic laboratory strain PAO1 revealed that type I RM system methyltransferases and their corresponding motifs altered virulence and oxidative stress response (Doberenz et al., 2017). The methylation recognition motif and methyltransferases of a clinical antiphagocytosis *P. aeruginosa* strain TBCF10839 confirmed that the methyltransferases deletion mutant had an important impact on improving bacterial virulence and Nitric Oxide Homeostasis (Han et al., 2022). Moreover, a single and sole methylation mutation on the *toxR* gene caused drastically different resistance against Ceftolozane/Tazobactam, Ceftazidime/Avibactam, and Piperacillin/Tazobactam in the isogenic pair of *P. aeruginosa* PB367 and PB350 l strains (W et al., 2020). These genetic and/or epigenetic elements are responsible for their notorious drug resistance.

In this study, SMRT sequencing was applied to gain a whole genome picture of *P. aeruginosa* clinical strain PA3 and insights into its DNA methylation patterns. By obtaining the methylation sites and sequences of the entire genome motif, bioinformatics analysis revealed 16 meaningful genes with methylation sites in their coding regions. Though the methyltransferases of these motifs need to be further identified, our findings provide a fundamental basis for further studies against *P. aeruginosa* infections.

## 2 Materials and methods

### 2.1 Bacterial storage and growth

The *P. aeruginosa* PA3 strain was maintained in the laboratory of the Department of Microbiology, Army Medical University. *P. aeruginosa* strains were stored in our laboratory at −80°C in glycerol. *P. aeruginosa* was cultured aerobically in Luria-Bertani (LB) medium (5 g yeast extract, 10 g tryptone, 10 g NaCl perliter) at 37°C for all experiments (Lu et al., 2015).

### 2.2 DNA extraction and SMRT sequencing

The PA3 genomic DNA was extracted and purified from the stationary phase cultures grown in LB broth using a DNeasy PowerClean Pro Cleanuo Kit (Shanghai Biotechnology Corporation). Approximately 10 μg purified PA3 genomic DNA was subjected to SMRT sequencing at Shanghai Biotechnology Corporation (SHBIO, Shanghai, China) using PacBio RSII (Pacific Biosciences). PA3 genomic DNA was fragmented using g-TUBE (Covaris, U.S.), and the 8-12k fragment was purified using AMPure magnetic beads (Beckman Coulter, U.S.). SMRTbell template library containing 10kb DNA fragments was prepared using DNA Template Prep Kit 2.0 (Pacific Biosciences, U.S.). Qubit^®^ 2.0 Fluorometer was used to measure the concentration of the SMRTbell template library, and Agilent2100 was used to measure the size of the library. SPAdes-3.5.0 software was used to accomplish *de novo* assembly.

### 2.3 Sequence analysis and genome annotation

The PA3 genome was annotated using the NCBI Prokaryotic Genome Automatic Annotation Pipeline (PGAAP) (http://www.ncbi.nlm.nih.gov/genome/annotation_prok/), which included genes, proteins, rRNAs, and tRNAs (Angiuoli et al., 2008). The genome data has been uploaded to the NCBI Genebank (CP061034.1). The Restriction-Modification (RM) system was predicted in REBASE (http://rebase.neb.com/rebase/rebase.html) (Roberts et al., 2022). Horizontal Gene Transfer (HGT) was predicted by alien_hunter 1.7 (GS and J, 2006). VirSorter 2.2.3 was used to predict dsDNA and ssDNA virus genomes (phage) (Guo et al., 2021). Mobile genetic elements (MGEs) were detected by mobileOG-db (beatrix-1.6) (Brown et al., 2022). SignalP6.0(SignalP - 6.0 - Services - DTU Health Tech) was used to annotate whether the protein sequence contains a signal peptide structure (Teufel et al., 2022). TMHMM2.0 (TMHMM - 2.0 - Services - DTU Health Tech) was used to annotate whether the protein sequence contains a transmembrane structure (Sonnhammer et al., 1998). DNA base modification detection took the splicing result as the reference genome. Through the *in silico control* analysis method in the *RS_Modification_and_Motif_Analysis.1 process*, the positive and negative strand base modification information and possible motifs of each position on the genome were obtained (Yang and Scott, 2017).

### 2.4 Visualized analysis of the PA3 genome

The PA3 genome was shown using the Ring Image Generator (BRIG) and Basic Local Alignment Search Tool (BLAST) (http://brig.sourceforge.net/) (NF et al., 2011). The Predicted Prokaryotic Regulatory Protein Server predicted regulatory proteins (RPs) (http://www.p2rp.org/) (Barakat et al., 2013) and visualized using Proksee (Proksee - New). The TAfinder program was used to forecast type II toxin-antitoxin (T-A) (http://202.120.12.133/TAfinder/index.php) and visualized using BRIG. IslandViewer (http://www.pathogenomics.sfu.ca/islandviewer/) was used to examine genomic islands (Bertelli et al., 2017) and visualized using Proksee.

### 2.5 Comparative genomic analysis

The 20 complete *P. aeruginosa* genome sequences were compared through BlastN by using blast 2.2.29+ (ftp://ftp.ncbi.nlm.nih.gov/blast/) (Boratyn et al., 2013) and visualized by BRIG with 80% identity cut-off. The PA3 genome was used as a reference. Pseudomonas Genome Database (http://www.pseudomonas.com/) was used to get 16S rRNA sequences and 20 full *P. aeruginosa* genome sequences (Winsor et al., 2016). 16S rRNA sequences were subjected to multiple sequence alignments by using ClustalW (Hung et al., 2015) with default parameters, and phylogenetic trees were constructed and displayed by MEGA 11 (http://www.megasoftware.net/) (Tamura et al., 2021) with the neighbor-joining method (Som and Fuellen, 2009). EDGAR and BPGA1.3 were used for pan-genome analysis (Chaudhari et al., 2016) with default parameters. The findings of EDGAR and BPGA were merged to present the *P. aeruginosa* pan-genome. The EDGAR software platform (https://edgar3.computational.bio.uni-giessen.de/cgi-bin/edgar_login.cgi?cookie_test=1) was used to create the Venn diagram (Aras et al., 2015). Since we focus more on epigenetic analysis, the description and discussion of comparative genome analysis were mainly placed in the Result section.

### 2.6 Methylation analysis in SMRT sequencing data

DNA methylation and motif prediction were performed using the RS_Modification_and_Motif_Analysis.1 analysis from PacBio portal 2.3. Kinetic data generated during genome sequencing was utilized for methylation detection (Flusberg et al., 2010). The default quality value (QV) of 30 is routinely used as the threshold for initial analysis. Subsequently, we increased the QV threshold to 100 to exclude incorrect motifs that occurred when the QV value was set to the default (QV>30) for highly covered regions. The QV and motif base count change is presented in **Supplementary Figure 2**. The screening statistics for DNA methylation sites data were implemented using R version 4.3.0.

## 3 Results and analysis

### 3.1 Visualized PA3 general genomic features

**Figure 1:**
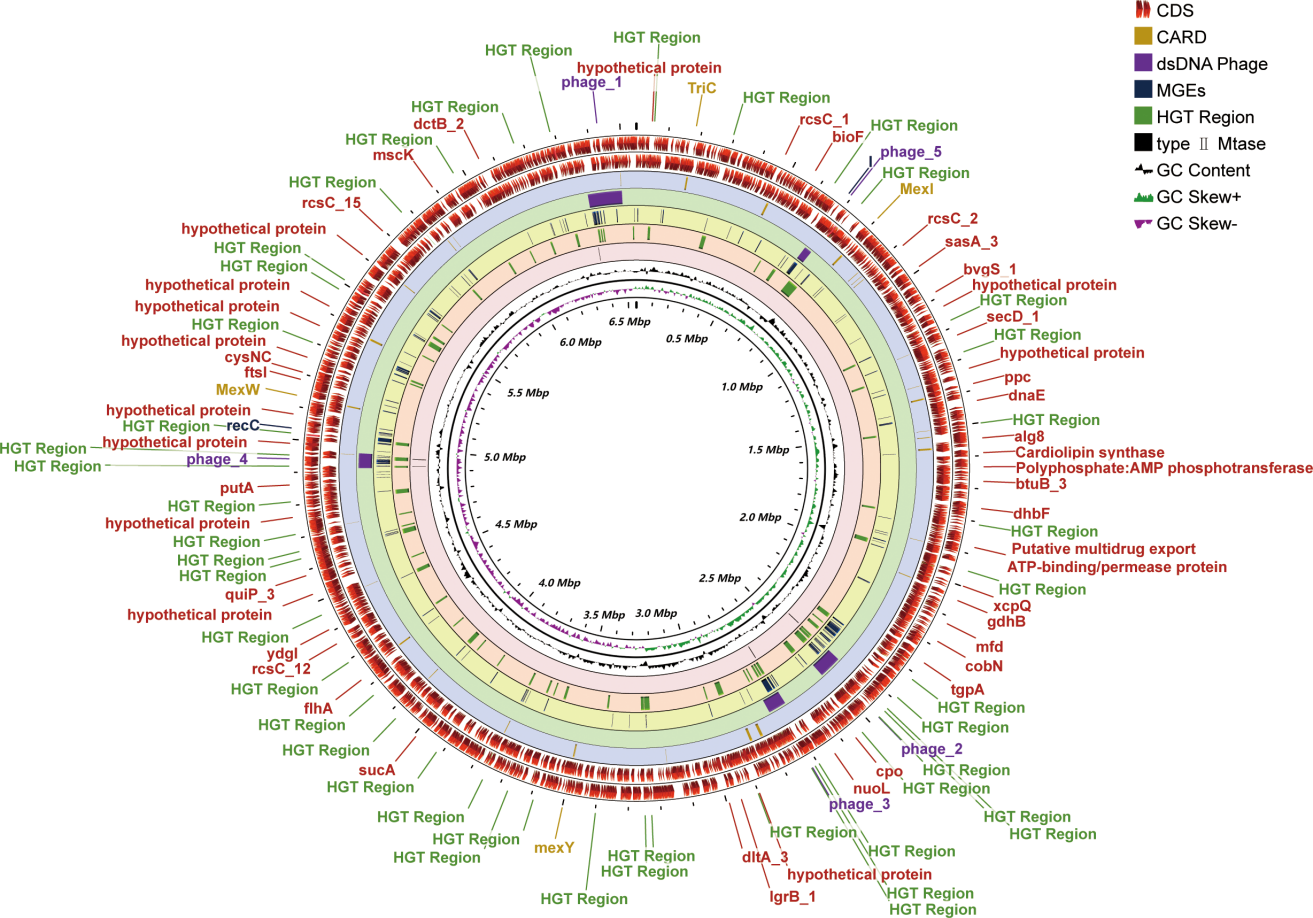
Genome-wide circular map of *P. aeruginosa* PA3. PA3 Genome Visualization Circle Diagram. In the outermost region, protein-coding regions (CDS) are indicated in red. From outside to inside, the outermost loop shows the CDS on the negative strand, followed by the CDS on the positive strand, the Comprehensive Antibiotic Resistance Database (CARD) resistance gene cite ds DNA phage sequence, MGEs, HGT region, putative MTase, GC content (black), and GC bias (purple/green).

One contig with a 432-fold sequencing coverage was found after the *de novo* assembly of the PA3 genome. 6,550,149 bp with 66.35% G+C, make up the entire PA3 genome (**Table 1**). A total of 6113 genes were annotated, including 5869 protein-coding genes, and 79 RNA genes. In addition, we also predicted 16 Pseudo Genes and 1 CRISPR (Clustered Regularly Interspaced Short Palindromic Repeats) array. VirSorter 2.2.3 predicted 5 putative dsDNA phages. Alien_hunter 1.7 predicts 60 horizontal transfer (HGT) regions. REBASE found 5 type II DNA methyltransferases predicted in the genome. In addition, mobileOG-db revealed 276 putative mobile gene elements (MGES) in the genome, including 53 IEs (integration/excision), 57 RRRs (replication/recombination/repair), 98 phages, 10 STDs (stability/transfer/defense), 58 transfers. These elements from different databases overlapped in the PA3 genome and are displayed in **Figure 1** for a global view.

**Table 1:** General genomic features of *P. aeruginosa* PA3.

Features	PA3
Accession	CP061034.1
Genome size(bp)	6,500,149
GC content	66.50%
Gene	6113
CDS	6034
Gene (coding)	5869
RNA	79
rRNA	16
5S rRNA	4
16S rRNA	4
30S rRNA	4
tRNA	63
ncRNA	4
Pseudogene	165
CRISPR Arrays	1

CDS, coding sequence;

rRNA, ribosomal RNA;

tRNA, transfer RNA;

ncRNA, non-coding RNA;

CRISPR, Clustered Regularly Interspaced Short Palindromic Repeats.

#### 3.1.1 Virulence factors

11 types of virulence factors of PA3 are predicted, including adherence, antimicrobial activity, anti-phagocytosis, biosurfactant, enzyme, iron uptake, protease, quorum sensing, regulation, secretion system, and toxin (Details in **Table 2** and **Supplementary Table 1**). Compared with the standard *P. aeruginosa* strain PAO1, PA3 has the same virulence factors except for *fimT* (encoding type IV pilus biosynthesis), five Phenazines biosynthesis genes (*phzC2, phzD2, phzE2, phzF2, phzG1*), and *pscE* (encoding the *P. aeruginosa* TTSS (Type Three Secretion System) secretion system).

**Table 2:** Virulence factors of *P. aeruginosa* PA3.

VF class	Virulence	Related genes	PA3	PAO1	Differential gene
Adherence(4 Item)					
Adherence	Type IV pili twitching motility related proteins	10	+	+	
Adherence	Type IV pili biosynthesis	24	23	+	*fimT*
Adherence	LPS O-antigen (P. aeruginosa)	1	+	+	
Adherence	Flagella	46	+	+	
Antimicrobial activity (1 Item)					
Antimicrobial activity	Phenazines biosynthesis	17	12	+	*phzC2, phzD2* *phzE2, phzF2* *phzG1*
Antiphagocytosis (2 Items)					
Antiphagocytosis	Alginate regulation	12	+	+	
Antiphagocytosis	Alginate biosynthesis	13	+	+	
Biosurfactant (1 Item)					
Biosurfactant	Rhamnolipid biosynthesis	3	+	+	
Enzyme (4 Items)					
Enzyme	Phospholipase D	1	+	+	
Enzyme	Phospholipase C	1	+	+	
Enzyme	Non-hemolytic phospholipase C	1	+	+	
Enzyme	Hemolytic phospholipase C	1	+	+	
Iron uptake (6 Items)					
Iron uptake	Yersiniabactin	9	–	–	
Iron uptake	Pyoverdine receptors	1	+	+	
Iron uptake	Pyoverdine	16	+	+	
Iron uptake	Pyochelin receptor	1	+	+	
Iron uptake	Pyochelin	10	+	+	
Iron uptake	Achromobactin biosynthesis and transport	8	–	–	
Protease (3 Items)					
Protease	Protease IV	1	+	+	
Protease	Elastase	2	+	+	
Protease	Alkaline protease	1	+	+	
Quorum sensing (4 Items)					
Quorum sensing	N-(butanoyl)-L-homoserine lactone QS system	2	+	+	
Quorum sensing	N-(3-oxo-hexanoyl)-Lhomoserine lactone QS system	2	–	–	
Quorum sensing	N-(3-oxo-dodecanoyl)-L-homoserine lactone QS system	2	+	+	
Quorum sensing	Acylhomoserine lactone synthase	1	+	+	
Regulation (1 Item)					
Regulation	GacS/GacA two-component system	2	+	+	
Secretion system (6 Items)					
Secretion system	P. syringae TTSS effectors	81	–	–	
Secretion system	P. syringae TTSS	32	–	–	
Secretion system	P. aeruginosa TTSS translocated effectors	4	3	3	
Secretion system	P. aeruginosa TTSS	36	35	+	*pscE*
Secretion system	Hcp secretion island-1 encoded type VI secretion system (H-T6SS)	21	+	+	
Secretion system	Harpins, pilus-associated proteins and other candidate TTSS helpers	6	–	–	
Toxin (8 Items)					
Toxin	TccC-type insecticidal toxins	1	–	–	
Toxin	Phytotoxin syringopeptin	3	–	–	
Toxin	Phytotoxin syringomycin	7	–	–	
Toxin	Phytotoxin phaseolotoxin	21	–	–	
Toxin	Phytotoxin coronatine	20	–	–	
Toxin	Hydrogen cyanide production	3	+	+	
Toxin	Exotoxin A (ETA)	1	+	+	
Toxin	Exolysin	2	–	–	

VF class, types of virulence factors;

Differential gene: differential gene between PAO1 and PA3.

#### 3.1.2 Regulatory proteins

**Figure 2:**
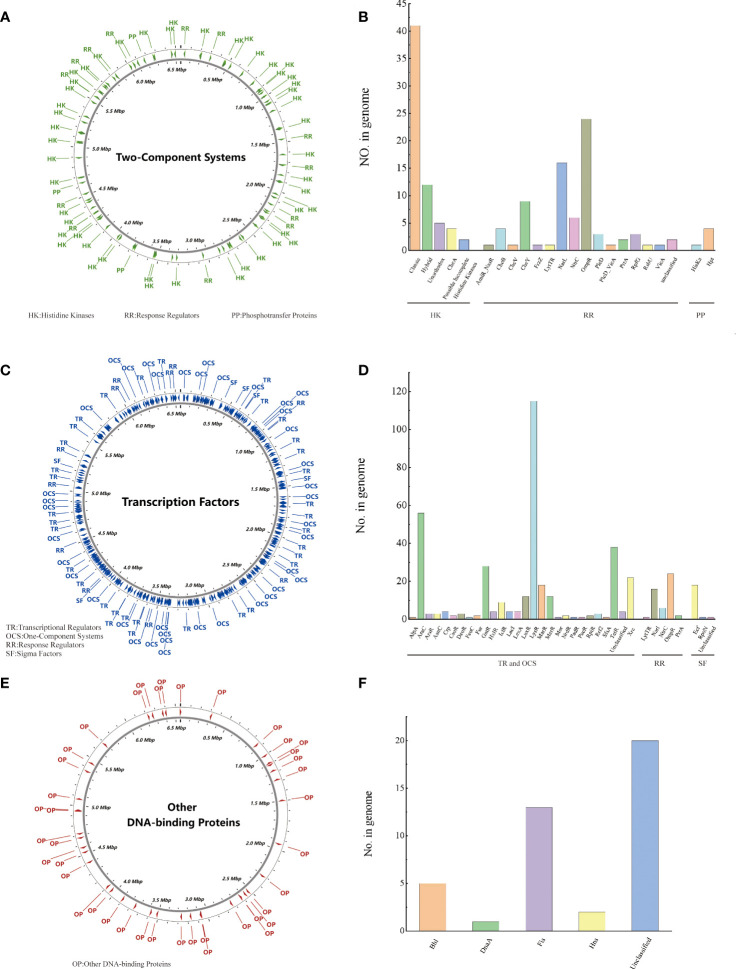
Regulatory proteins of *P. aeruginosa* PA3. **(A)** Distribution of TCS in the PA3 genome. **(B)** Detailed number and species of TCS proteins encoded by PA3. **(C)** Distribution of TFs in the PA3 genome. **(D)** Detailed number and species of TFs encoded by PA3. **(E)** Distribution of ODP in the PA3 genome. **(F)** Detailed quantities and types of ODPs encoded by PA3.

Regulatory proteins (RPs) cause bacteria to adapt to changes in their environment. PA3 can be predicted to encode 614 regulatory proteins. Two-Component Systems (TCS) of PA3 contain 64 histidine kinases, 76 response regulators, and 5 phosphotransfer proteins, which are evenly distributed in the genome (**Figures 2A, B**). 181 transcriptional regulators, 175 single-component systems, 49 response regulators, and 23 sigma factors were among the transcription factors (TFs) of PA3 (**Figures 2C, D**). Along with these 41 additional DNA-binding proteins (ODPs, other DNA-binding proteins), PA3 also encodes 20 unclassified ODPs, *Bhl*, *DnaA*, *Fis*, and *Hns* (**Figures 2E, F**). **Supplementary Tables 2**-**4** show detailed information about the RPs of PA3 for further research.

#### 3.1.3 Secretory proteins

**Figure 3:**
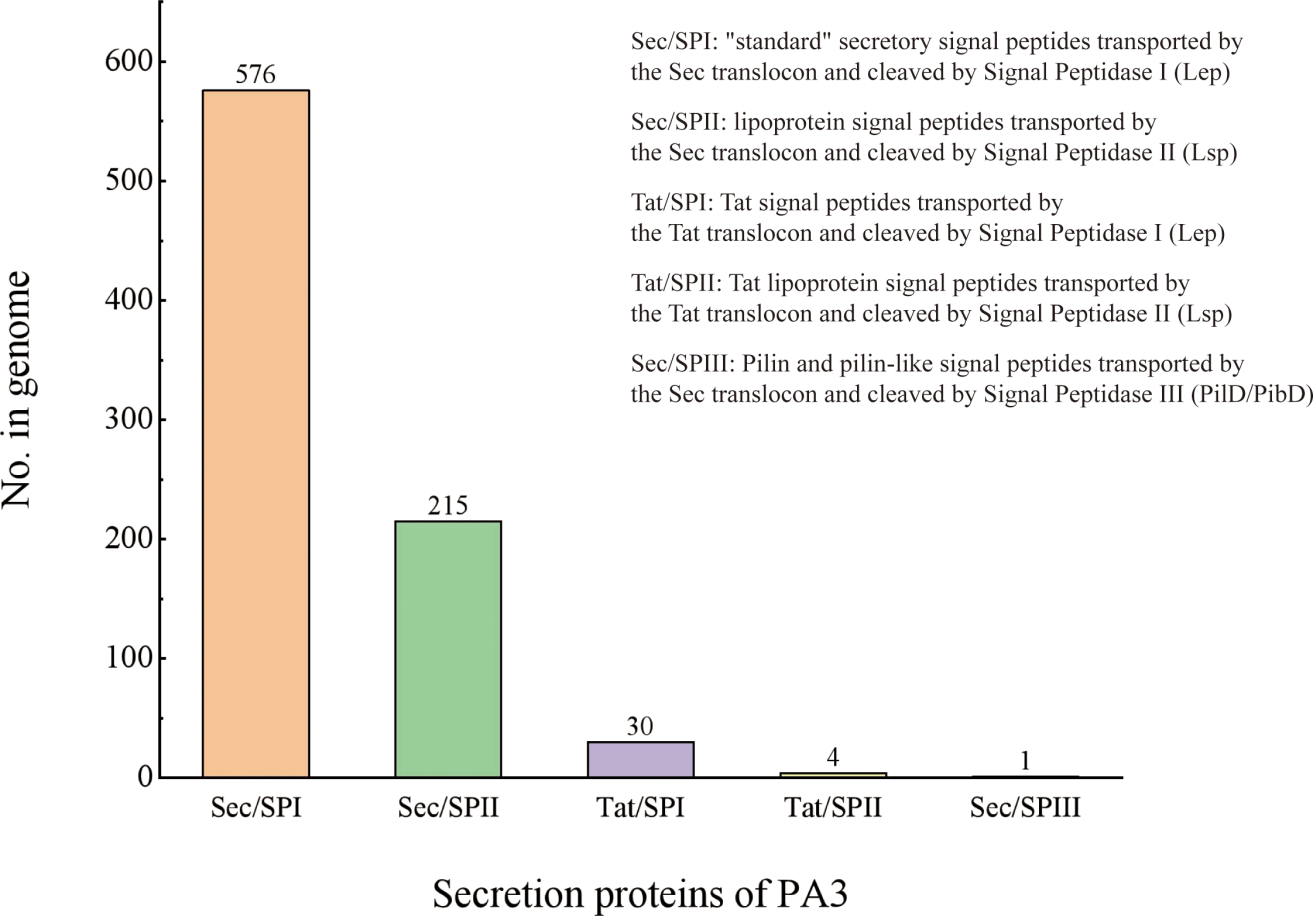
Secretory proteins of P. aeruginosa PA3. PA3 contains 576 Sec/SPI [“standard” secretory signal peptides transported by the Sec translocon and cleaved by Signal Peptidase I (Lep)], 215 Sec/SPII [lipoprotein signal peptides transported by the Sec translocon and cleaved by Signal Peptidase II (Lsp)], 30 Tat/SPI [Tat signal peptides transported by the Tat translocon and cleaved by Signal Peptidase I (Lep)], 4 Tat/SPII [Tat lipoprotein signal peptides transported by the Tat translocon and cleaved by Signal Peptidase II (Lsp)], 1 Sec/SPIII [Pilin and pilin-like signal peptides transported by the Sec translocon and cleaved by Signal Peptidase III (PilD/PibD)].

Secretory Protein refers to a protein that is secreted to function outside the cell after being synthesized in the cell. The secretory proteins of PA3 were detected when the protein contains signal peptide structures (annotated by SignalP) without transmembrane structures (annotated by TMHMM). A total of 895 protein sequences containing signal peptides were detected, 69 of which were predicted to have transmembrane helical regions. Thus, we found 826 secretory proteins in the PA3 genome (see **Figure 3** and **Supplementary Table 5** for details).

#### 3.1.4 Type II toxin-antitoxin

**Figure 4:**
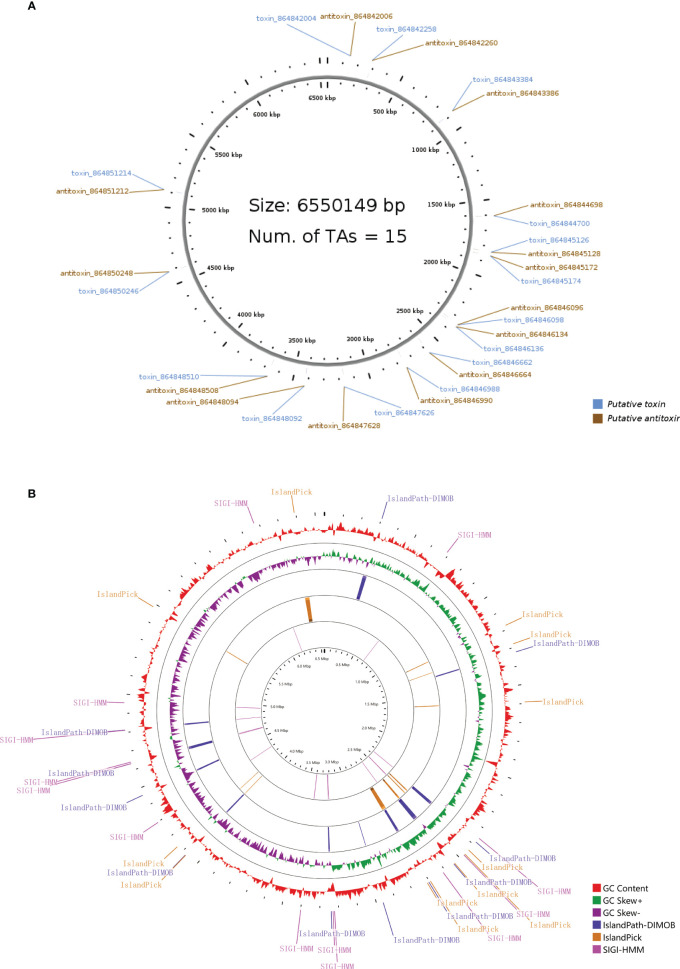
Type II T–A loci and genomic islands of *P. aeruginosa* PA3. **(A)** Distribution of 15 pairs of type II T–A loci in the PA3 genome. **(B)** Genomic islands in the PA3 genome. From outside to inside are GC content (red) and GC bias (purple/green) and three methods for predicting gene islands, IslandPath-DIMOB, IslandPick, SIGI-HMM (Single Island Genetic Island Hidden Markov Model).

The type II toxin-antitoxin (TA) system performs several critical cellular activities, including phage defense, biofilm development, persistence, and virulence (Kamruzzaman et al., 2021). We found 15 type II TA pairs in the PA3 genome (**Figure 4A**). 6 type II TA pairs were predicted by BLASTP method and 9 type II TA pairs were predicted by HMM method. When *P. aeruginosa* strains, PAO1, PA7, and PA14 have 13, 19, and 17 type II TA pairs, respectively (detailed information in **Supplementary Table 6**). Based on the comparison of the conserved domains of different *P. aeruginosa* strains, we found a unique antitoxin domain MerR in PA3, encoded by gene *orf4389*. The MerR family transcriptional regulators have been shown to mediate responses to environmental stress (Kamruzzaman et al., 2021) suggesting a different environmental tolerance of PA3 from other *P. aeruginosa* strains.

#### 3.1.5 Genomic islands

Genomic islands are groups of genes in bacterial genomes that have been acquired by horizontal gene transfer (Juhas et al., 2009). With SMRT technique, we found 36 genomic islands spanning 345 genes in PA3 (**Figure 4B**). Their main concentration distribution in the genome is the same as that of MGEs, HGT region, and phage gene in **Figure 1**. The largest genomic island was 39.7 kb in length (from 6,371,760 kb to 6,411,492 kb), named PA3_G36, and consisted of 51 genes. The PA3_G36 genomic island encodes a type II RM system methyltransferase, a Soluble lytic murein transglycosylase. See **Supplementary Table 7** for specific information on the PA3 genomic island.

### 3.2 Comparative genomic analysis of *P. aeruginosa*

**Figure 5:**
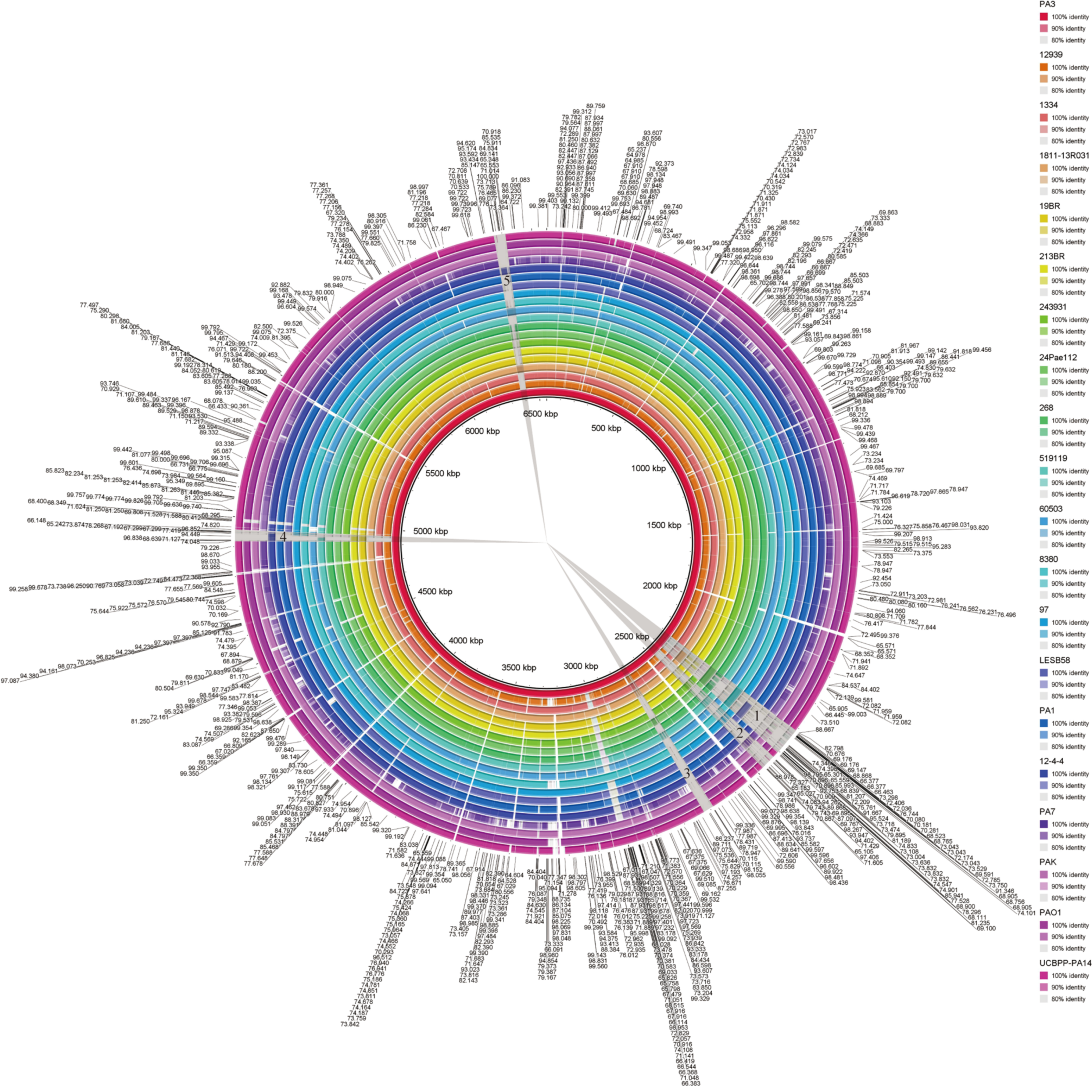
BLAST comparison of the complete genome of *P. aeruginosa* PA3 with 19 other *P. aeruginosa* species. Figure legends of the 20 P*. aeruginosa* strains are shown from innermost to outermost. Identity is indicated by color.

Visual genome comparisons are necessary to help identify genotypic differences among closely related bacteria. 20 strains of *P. aeruginosa* were compared by BRIG and visualized. These results showed that the *P. aeruginosa* genome sequences were very similar, with most of the compared genomic regions showing more than 85% identity compared with the PA1 genome (**Figure 5**). However, there are still several relatively large genomic regions (over 30 kb in length) unknown (less than 85% identity), as indicated by numbers 1 to 5 in **Figure 4**. These regions from long to short are 94.70, 52.16, 36.29, 37.86 and 39.80 kb in length, respectively.

#### 3.2.1 Phylogenetic analysis

**Figure 6:**
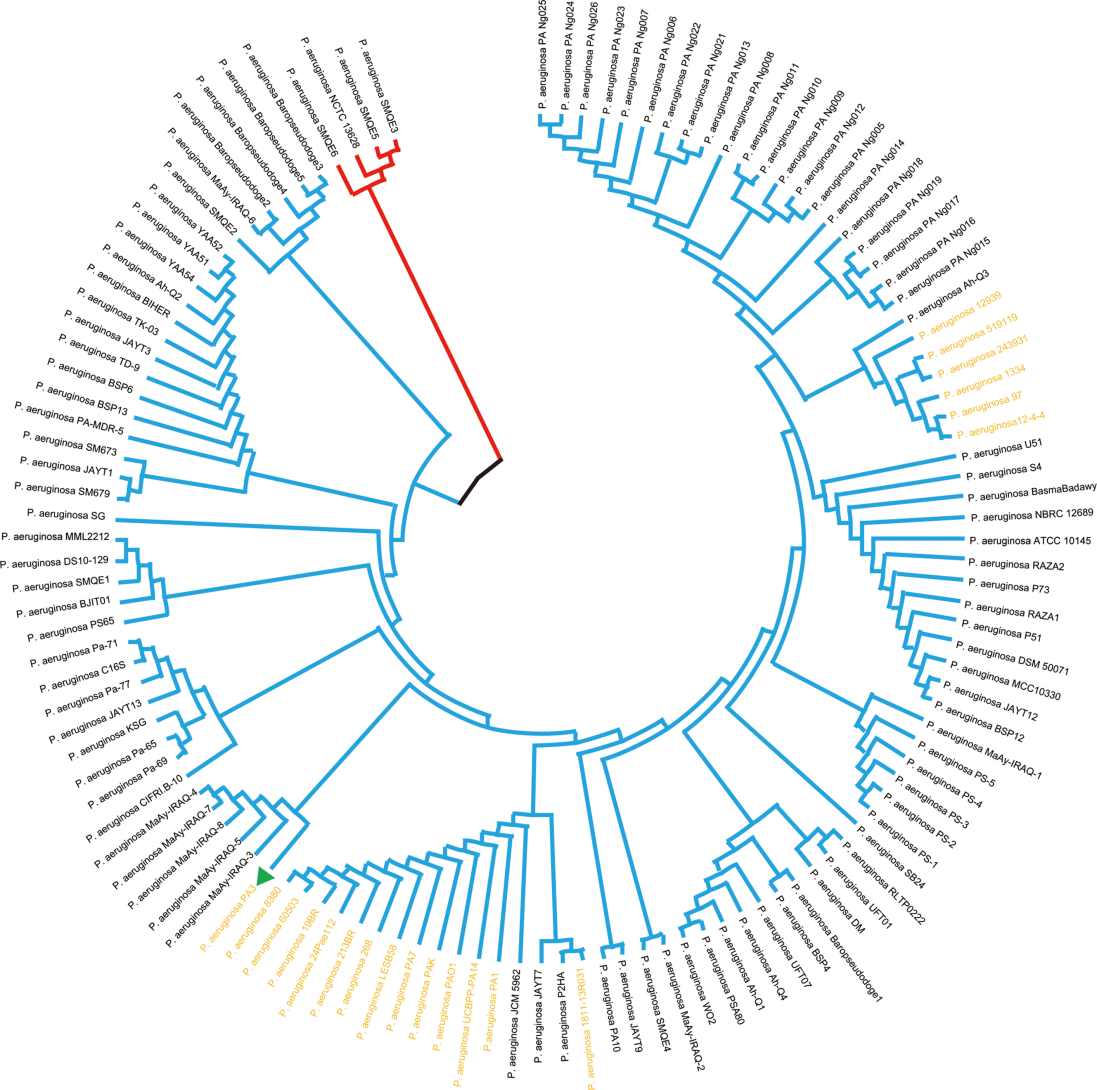
Phylogenetic tree of *P. aeruginosa* based on 16S rRNA. Phylogenetic relationships of 126 strains of *P. aeruginosa* (showing only topological structure). The two subgroups are shown in red and blue branches, respectively. The 20 comparative *P. aeruginosa* strains in **Figure 4** are shown in bold yellow font.

Prokaryotic 16S ribosomal RNA (rRNA) sequences are widely used in environmental microbiology and molecular evolution as reliable markers for microbial taxonomy and phylogenetic analysis(Adhikari et al., 2015; Yang et al., 2016). This slight difference of 16S rRNA sequences indicated discriminable evolution processes of 126 P*. aeruginosa* species strains (including PA3). PA3 is closely related to *P. aeruginosa* 60503 and *P. aeruginosa* 8380 (**Figure 6**). The original phylogenetic tree with relative genetic distances is shown in **Supplementary Figure 1**.

#### 3.2.2 Pan-genome analysis

**Figure 7:**
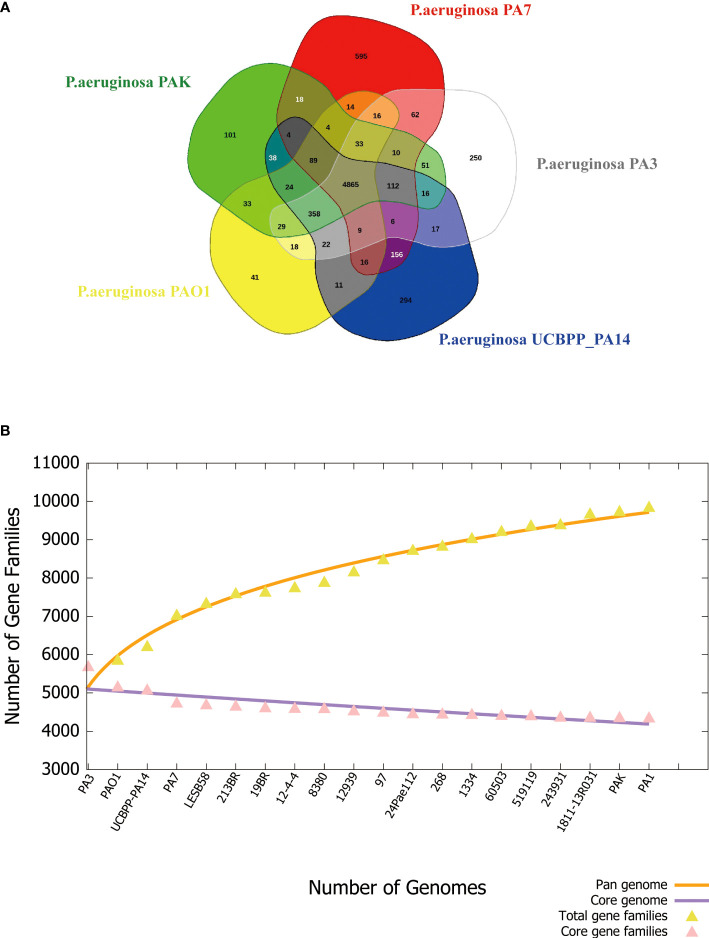
Phylogenetic tree of *P. aeruginosa* based on 16S rRNA. **(A)** Venn diagram of five *P. aeruginosa* strains showing the number of core genes, accessory genes, and their unique genes. **(B)** Pan- and core-genomes of 20 strains of *P. aeruginosa* with complete genomes.

The pan-genome can be divided into 3 parts, core genes, accessory genes, and unique genes. The core genome is the genes shared by all strains involved in basic biological processes such as gene expression, energy production, amino acid metabolism, etc. Accessory genes refer to genes present in specific strains, which are related to the diversity of the species and confer a competitive advantage on the individual. Unique genes exist only in a certain strain and are usually related to the unique phenotypic characteristics of the strain, such as adaptability to a specific environment, or unique pathogenicity (Yang et al., 2016). PA3 shares 4,865 core genes with four additional common *P. aeruginosa* strains, including PAO1, PA7, PAK, and UCBPP-PA14, according to the Venn diagram (**Figure 7A**). A total of 2,449 accessory genes were predicted, with 1,281 of these genes being unique. **Figure 7B** shows that the *P. aeruginosa* pan-genome has at least 4,300 core genes and 5,500 auxiliary genes. With the increase in genome number, the pan-genome size of *P. aeruginosa* also increased, suggesting that *P. aeruginosa* has a strong ability to integrate exogenous DNA and a high level of polymorphism.

### 3.3 Epigenetic analysis of *P. aeruginosa* PA3

We found 2032 m6A DNA methylation sites of P. aeruginosa PA3. Moreover, 1 motif (C**A**TNNNNNNNTCCT/AGG**A**NNNNNNNATG) was predicted and scored (**Table 3**). We screened out all C**A**TNNNNNNNTCCT/AGG**A**NNNNNNNATG sequences with m6A in the PA3 genome and found 16 corresponding genes with such m6A sites, which have high scores and coverage and we believe may have a potential role in gene regulation. (**Table 4**).

**Table 3:** Predicted motif sequences of *P. aeruginosa* PA3.

Motifstring	Centerpos	Modificationtype	Fraction	nDetected	nGenome	Meanscore	Objectivescore
C**A**TNNNNNNNTCCT	1	m6A	0.990272	1018	1028	246.3144	248551.7
AGG**A**NNNNNNNATG	3	m6A	0.986381	1014	1028	237.7525	238124.1

Motifstring, detected motif sequence for this site;

Centerpos, position in the motif of modification (0-based), methylated base sites are indicated in bold;

Fraction, the percent of the time this motif is detected as modified in the genome;

nDetected, number of instances of this motif that are detected as modified;

nGenome, number of this motif in the genome;

Meanscore, the average modification score of the motif;

Objectivescore, score of this motif in the motif finder algorithm.

(R = G or A; Y = C or T; M = A or C; K = G or T; W = A or T; B = not A (C or G or T); D = not C (A or G or T); H = not G (A or C or T); V = not T (A or C or G); N = A or C or G or T).

**Table 4:** m6A sites and their corresponding genes of *P. aeruginosa* PA3.

Type	Start	End	Score	Strand	Name	Product
m6A	27749	27749	418	+	*EBO27_00130*	phospholipase C
m6A	120436	120436	408	+	*EBO27_00535*	DUF4150 domain-containing protein
m6A	242050	242050	412	+	*EBO27_01100*	malonate decarboxylase holo-ACP synthase
m6A	416658	416658	343	+	*EBO27_01900*	TetR/AcrR family transcriptional regulator
m6A	816157	816157	421	+	*EBO27_03840*	response regulator transcription factor
m6A	1287374	1287374	336	+	*EBO27_06050*	aromatic amino acid transporter
m6A	5691946	5691946	345	+	*purH*	bifunctional phosphoribosylaminoimidazolecarboxamide formyltransferase/IMP cyclohydrolase
m6A	5941968	5941968	357	+	*phaZ*	poly(3-hydroxyalkanoate) depolymerase
m6A	6408802	6408802	551	+	*EBO27_29895*	DUF2786 domain-containing protein
m6A	6418839	6418839	362	+	*EBO27_29940*	EamA family transporter
m6A	402746	402746	422	–	*ilvD*	dihydroxy-acid dehydratase
m6A	659788	659788	352	–	*pdxA*	4-hydroxythreonine-4-phosphate dehydrogenase PdxA
m6A	659980	659980	341	–	*pdxA*	4-hydroxythreonine-4-phosphate dehydrogenase PdxA
m6A	1020212	1020212	360	–	*EBO27_04790*	alpha/beta fold hydrolase
m6A	2115743	2115743	338	–	*lexA*	transcriptional repressor LexA
m6A	5582718	5582718	343	–	*carB*	carbamoyl-phosphate synthase large subunit

m6A, N6-methyladenosine.

#### 3.3.1 Restriction-modification system predicted

Based on REBASE analysis with PA3 methylation information uploaded, five MTase genes were predicted in the PA3 genome belonging to type II RM system named M.PaesPA3ORF11530P, M.PaesPA3ORF12905P, M.PaesPA3ORF23015P, M.PaesPA3ORF23200P, and M.PaesPA3ORF29655P (**Table 5**). The methylation motifs recognized by these five MTases were not predicted in REBASE. The predicted methylation motifs by SMRT were uploaded to the REBASE. Only C ^m6^**A**TNNNNNNNTCCT/AGG ^m6^**A**NNNNNNNATG could be detected. However, this motif was only known to belong to type I RM systems and could not be identified by any known enzymes.

**Table 5:** REBASE predicted genes of *P. aeruginosa* PA3.

Type	Gene	Name	Predicted Rec Seq	Contig	Coordinates
II	*M*	*M.PaesPA3ORF11530P*	—	—	2,430,946-2,432,943
II	*M*	*M.PaesPA3ORF12905P*	—	—	2,689,047-2,690,957
II	*M*	*M.PaesPA3ORF23015P*	—	—	4,919,963-4,920,766
II	*M*	*M.PaesPA3ORF23200P*	—	—	4,952,798-4,954,633
II	*M*	*M.PaesPA3ORF29655P*	—	—	6,371,839-6,372,633

II: type II RM modification system;

M: methyltransferase;

Predicted Rec Seq: predicted recognition sequence.

#### 3.3.2 Screened m6A sites and genes of PA3

**Figure 8:**
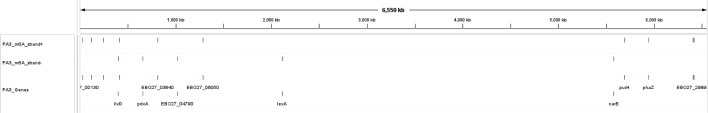
The overview of 16 m6A methylation sites and their genes in *P. aeruginosa* PA3. The three tracks include 10 m6A sites on the positive strand, 6 sites on the negative strand, and their corresponding 16 genes.

The distributions of 16 genes with C ^m6^**A**TNNNNNNNTCCT/AGG ^m6^**A**NNNNNNNATG methylation sites were shown in **Figure 8**. These genes contain multiple functions and the most noteworthy genes are *purH*, *phaZ*, and *lexA*. Gene *purH* is *a bi*functional phosphoribosyl aminoimidazole carboxamide formyltransferase/IMP cyclohydrolase, and plays a key role in maintaining folate in cells. The last two steps of *de novo* purine biosynthesis are catalyzed by *purH*. Gene *phaZ* is a depolymerase of polyhydroxyalkanoic acid (PHA), a storage substance synthesized by many prokaryotes. The transcriptional repressor *lexA* plays an important role in bacterial DNA damage repair.

## 4 Discussion

The benefits of SMRT sequencing are high throughput, quick turnaround time, high consistency accuracy, and a minimal quantity of DNA samples (Schadt et al., 2010). Moreover, it can accurately detect the modification of a single base, which is very suitable for bacteria sequencing and methylation analysis (Zautner et al., 2015). The study of the bacterial methylome has been revolutionized by introducing technologies capable of detecting m4C, 5-methylcytosine (m5C), and m6A at a genome-wide scale and single-nucleotide resolution. Thus resequencing projects of *P. aeruginosa* PA3 is necessary and advantageous to provide a better understanding of the number and diversity of genetics and epigenetics instead of the outdated sequencing techniques and mis-curated genome.

The majority of the studied *P. aeruginosa* genome regions are compatible with the PA3 genome. Portions of the PA3 genome that differ from those of other *P. aeruginosa* species are regions of horizontal gene transfer (also known as MGES) as also reported in our previous research on *P. aeruginosa* PA1 (Li et al., 2016). In this study, 336 putative mobile gene elements (MGES) carrying resistance genes or virulence factors were revealed in the *P. aeruginosa* PA3 genome. Infections caused by *P. aeruginosa* are attributed to virulence factors, such as flagella, alginate regulation factor, and phospholipase D. However, PA3 has the same virulence factors except for *fimT* (type IV pilus biosynthesis), 5 Phenazines related genes (*phzC2, phzD2, phzE2, phzF2, phzG1*), and *pscE* (TTSS secretion system). The difference in the virulence factors between *P. aeruginosa* PA3 and other *P. aeruginosa* strains suggests that the expression of virulence factors is dynamic and evolving (VV et al., 2022), which may explain the distinctive virulence characteristics between them. For instance, it has been revealed that type IV pili in the *fimT* mutant are not fully functional, and *fimT* mutation definitively affected bacterial function, including surface motility, gene expression, and virulence (Taguchi and Ichinose, 2011). The major component of the *P. aeruginosa* T3S needle is the PscF protein. The *pscE* knockout strain of *P. aeruginosa* lacks PscF protein and is therefore non-cytotoxic (Quinaud et al., 2005). Since a variety of MGEs or HGT regions have been isolated from clinical drug-resistant strains PA3, more in-depth research can be carried out on them.

From the epigenetic view, epigenomic changes were associated with bacterial phenotypes including antimicrobial resistance (Huang et al., 2020). Here we found 1 methylation motif in PA3. Whether there is a recognized relationship between the motif and 5 MTase has not been experimentally verified. The Mbase predicted by REBASE does not match our predicted motif, suggesting that the gene responsible for encoding this part of Mbase may be carried by a temperate bacteriophage. The identification of PA3 methyltransferases and the motifs they recognize play an important role in regulating gene methylation, which also provides new antibacterial insights. The DNA methylation pattern of PA3 was illustrated including one major methylation modification type m6A. Moreover, 16 methylated sites with high scores and coverage, which we believe may have a potential role in gene regulation were picked. Among these, *purH, phaZ*, and *lexA* are of great significance playing an important role in the drug resistance and biological environment adaptability of PA3.

Exogenous glutamine can enhance the killing of ampicillin against multi-drug-resistant *P. aeruginosa* and other fungi. In a mouse model of urinary tract infection, glutamine enhanced the lethality of ampicillin and was effective against systemic infection caused by *P. aeruginosa*. Exogenous glutamine stimulates the influx of ampicillin, accumulating intracellular antibiotic concentrations above tolerated levels. The pathway is dependent on the genes/proteins CpxA/R, OmpF, *purF*, *purD*, *purL*, *purE*, *purB*, *purH*, and *purE* (Zhao et al., 2021). Loss of *purH* affects the pathway through which exogenous glutamine regulates membrane permeability and significantly increases antibiotic resistance in antibiotic-susceptible strains (Zhao et al., 2021). Therefore, we believe that the methylation of specific sites on *purH* through specific methyltransferases down-regulates the expression of *purH* and therefore increases the multiple antibiotics resistance of PA3 (**Supplementary Table 8**).

The synthesis of polyhydroxyalkanoic acid (PHA) is controlled by an integrated polymerization-depolymerization system of polymerases (PhaC1 and PhaC2) and depolymerase (PhaZ). It has been found that the *P. aeruginosa* PA14 *phaZ* mutant accumulated about 300% more PHA than the PA14 wild-type strain (Choi et al., 2011). Likewise, the inactivation of phaZ in *Pseudomonas putida* KT2442 (Cai et al., 2009) and *Pseudomonas fluorescens* BM07 (Choi et al., 2010) strains resulted in increased PHA accumulation. *Pseudomonas* In stress tolerance and biofilm formation, PHA-negative mutants establish more complex and differentiated biofilms than wild-type, which is thought to adapt to starvation stress due to the lack of ability to reserve PHA. The PHA-negative mutant was more heat sensitive than the non-mutant strain (Pham et al., 2004). The methylation site of *PhaZ* gene indicates a control mechanism regulating *P. aeruginosa’s* response to heat and other environmental changes.

Gene *lexA* has been extensively researched for its prodigious role in bacterial DNA damage repair. LexA inhibits the expression of SOS genes which is important for bacteria to respond to DNA damage. When DNA damage occurs, LexA is cleaved through complex signal transduction to ensure all genes involved in the SOS response are fully expressed (Kreuzer, 2013). This essential transcription factor also regulates the induction of phages, the migration of pathogenicity islands, and the production of virulence factors and bacteriocins (Fornelos et al., 2016). Moreover, the research of *lexA* also involves anti-drug resistance and biofilm formation (Cirz et al., 2006). And it was predicted that *lexA* may play an inhibitory role in motility function (Chellappa et al., 2013). Here we revealed the methylation site in *lexA*. Through the methylation of *lexA*, the above-mentioned suppression of DNA damage repair, biofilm formation, and drug resistance can be removed, which increases the competitiveness and adaption of PA3, which requires further phenotype analysis experiments to confirm.

This study has its limitation of no experimental verification. The bioinformatics analysis can integrate existing literature and database information, reveal the association between DNA methylation and phenotype characteristics in *P. aeruginosa*, and provide theoretical guidance for subsequent experimental designs. Future studies will combine experimental verification and pure bioinformatics analysis to investigate the specific mechanisms of methylation regulation in bacterial adaptability and competitiveness, which is going to be reported in our next study.

In conclusion, this study mapped the genome of a clinical *P. aeruginosa* strain PA3 with third-generation sequencing SMRT. The genomic features were cross-annotated through several fully developed and comprehensive databases. The DNA methylation pattern of PA3 was illustrated including one major methylation modification type m6A. Moreover, 16 methylated sites with high scores and coverage, which we believe may have a potential role in gene regulation were picked. Among these, *purH*, *phaZ*, and *lexA* are of great significance playing an important role in the drug resistance and biological environment adaptability of PA3, and the targeting of these genes may benefit further antibacterial studies.

## Data availability statement

The datasets used and/or analyzed during the current study areavailable from the corresponding author upon reasonable request.

## Ethics statement

This is an original research article on bacteria sequencing data. For this type of study, the requirement for ethics approval is waived by the Medical Ethics Committee of the Second Affiliated Hospital (Xinqiao Hospital) of Army Medical University, PLA.

## Author contributions

ZL and XZhou contributed equally to this work. ZY and YT contributed equally to this work. All authors contributed to the article and approved the submitted version.

## Supplementary material

Supplementary Figure 1 The original phylogenetic tree of *P. aeruginosa* PA3.

Supplementary Figure 2 The quality value (QV) and motif base count change of *P. aeruginosa* PA3.

Supplementary Table 1 Detailed list of virulence factors of *P. aeruginosa* PA3.

Supplementary Table 2 Two-component systems of *P. aeruginosa* PA3.

Supplementary Table 3 Transcription factors of *P. aeruginosa* PA3.

Supplementary Table 4 Other DNA-binding proteins of *P. aeruginosa* PA3.

Supplementary Table 5 Secretory proteins of *P. aeruginosa* PA3.

Supplementary Table 6 Type II toxin-antitoxin (TA) of *P. aeruginosa* PA3.

Supplementary Table 7 Genomic islands of *P. aeruginosa* PA3.

Supplementary Table 8 Drug sensitive tests of wide type PA3.

## References

[B1] Adhikari A. Nandi S. Bhattacharya I. Roy M. D. Mandal T. Dutta S. (2015). Phylogenetic analysis based evolutionary study of 16S rRNA in known Pseudomonas sp. Bioinformation 11 (10), 474–480. doi: 10.6026/97320630011474 26664032 PMC4658646

[B2] Alikhan N. F. Petty N. K. Zakour Ben N. L. Beatson S. A. (2011). BLAST Ring Image Generator (BRIG): simple prokaryote genome comparisons. BMC Genomics 12, 402. doi: 10.1186/1471-2164-12-402 21824423 PMC3163573

[B3] Angiuoli S. V. Gussman A. Klimke W. Cochrane G. Field D. Garrity G.. (2008). Toward an online repository of Standard Operating Procedures (SOPs) for (meta)genomic annotation. Omics: J. Integr. Biol. 12 (2), 137–141. doi: 10.1089/omi.2008.0017 PMC3196215 18416670

[B4] Anton B. P. Roberts R. J. (2021). Beyond restriction modification: epigenomic roles of DNA methylation in prokaryotes. Annu. Rev. Microbiol. 75, 129–149. doi: 10.1146/annurev-micro-040521-035040 34314594

[B5] Aras K. Good W. Tate J. Burton B. Brooks D. Coll-Font J.. (2015). Experimental data and geometric analysis repository-EDGAR. J. Electrocardiol. 48 (6), 975–981. doi: 10.1016/j.jelectrocard.2015.08.008 26320369 PMC4624576

[B6] Ardui S. Ameur A. Vermeesch J. R. Hestand M. S. (2018). Single molecule real-time (SMRT) sequencing comes of age: applications and utilities for medical diagnostics. Nucleic Acids Res. 46 (5), 2159–2168. doi: 10.1093/nar/gky066 29401301 PMC5861413

[B7] Attar N. (2016). Bacterial genetics: SMRT-seq reveals an epigenetic switch. Nat. Rev. Microbiol. 14 (9), 546. doi: 10.1038/nrmicro.2016.122 27477301

[B8] Barakat M. Ortet P. Whitworth D. E. (2013). P2RP: a Web-based framework for the identification and analysis of regulatory proteins in prokaryotic genomes. BMC Genomics 14, 269. doi: 10.1186/1471-2164-14-269 23601859 PMC3637814

[B9] Bertelli C. Laird M. R. Williams K. P. Lau B. Y. Hoad G. Winsor G. L.. (2017). IslandViewer 4: expanded prediction of genomic islands for larger-scale datasets. Nucleic Acids Res. 45 (W1), W30–W35. doi: 10.1093/nar/gkx343 28472413 PMC5570257

[B10] Boratyn G. M. Camacho C. Cooper P. S. Coulouris G. Fong A. Ma N.. (2013). BLAST: a more efficient report with usability improvements. Nucleic Acids Res. 41 (Web Server issue), W29–W33. doi: 10.1093/nar/gkt282 23609542 PMC3692093

[B11] Brown C. L. Mullet J. Hindi F. Stoll J. E. Gupta S. Choi M.. (2022). mobileOG-db: a manually curated database of protein families mediating the life cycle of bacterial mobile genetic elements. Appl. Environ. Microbiol. 88 (18), e0099122. doi: 10.1128/aem.00991-22 36036594 PMC9499024

[B12] Cai L. Yuan M. Q. Liu F. Jian J. Chen G. Q. (2009). Enhanced production of medium-chain-length polyhydroxyalkanoates (PHA) by PHA depolymerase knockout mutant of Pseudomonas putida KT2442. Bioresour. Technol. 100 (7), 2265–2270. doi: 10.1016/j.biortech.2008.11.020 19103481

[B13] Chaudhari N. M. Gupta V. K. Dutta C. (2016). BPGA- an ultra-fast pan-genome analysis pipeline. Sci. Rep. 6, 24373. doi: 10.1038/srep24373 27071527 PMC4829868

[B14] Chellappa S. T. Maredia R. Phipps K. Haskins W. E. Weitao T. (2013). Motility of Pseudomonas aeruginosa contributes to SOS-inducible biofilm formation. Res. In Microbiol. 164 (10), 1019–1027. doi: 10.1016/j.resmic.2013.10.001 24125694

[B15] Choi M. H. Xu J. Gutierrez M. Yoo T. Cho Y. H. Yoon S. C. (2011). Metabolic relationship between polyhydroxyalkanoic acid and rhamnolipid synthesis in Pseudomonas aeruginosa: comparative ¹³C NMR analysis of the products in wild-type and mutants. J. Biotechnol. 151 (1), 30–42. doi: 10.1016/j.jbiotec.2010.10.072 21029757

[B16] Choi M. H. Xu J. Rho J. K. Zhao X. P. Yoon S. C. (2010). Enhanced production of longer side-chain polyhydroxyalkanoic acid with omega-aromatic group substitution in phaZ-disrupted Pseudomonas fluorescens BM07 mutant through unrelated carbon source cometabolism and salicylic acid beta-oxidation inhibition. Bioresour. Technol. 101 (12), 4540–4548. doi: 10.1016/j.biortech.2010.01.082 20153638

[B17] Cirz R. T. O’Neill B. M. Hammond J. A. Head S. R. Romesberg F. E. (2006). Defining the Pseudomonas aeruginosa SOS response and its role in the global response to the antibiotic ciprofloxacin. J. Bacteriol. 188 (20), 7101–7110. doi: 10.1128/JB.00807-06 17015649 PMC1636241

[B18] Cohen N. R. Ross C. A. Jain S. Shapiro R. S. Gutierrez A. Belenky P.. (2016). A role for the bacterial GATC methylome in antibiotic stress survival. Nat. Genet. 48 (5), 581–586. doi: 10.1038/ng.3530 26998690 PMC4848143

[B19] Dan R. Marin K. (2021). The epidemiology and pathogenesis and treatment of Pseudomonas aeruginosa infections: an update. Drugs 81 (18), 2117–2131. doi: 10.1007/s40265-021-01635-6 34743315 PMC8572145

[B20] Dimopoulos G. Akova M. Rello J. Poulakou G. (2020). Understanding resistance in pseudomonas. Intensive Care Med. 46 (2), 350–352. doi: 10.1007/s00134-019-05905-6 31960069 PMC7224039

[B21] Doberenz S. Eckweiler D. Reichert O. Jensen V. Bunk B. Spröer C.. (2017). Identification of a pseudomonas aeruginosa PAO1 DNA methyltransferase, its targets, and physiological roles. MBio 8 (1), e02312-16. doi: 10.1128/mBio.02312-16 PMC5358918 28223461

[B22] Eid J. Fehr A. Gray J. Luong K. Lyle J. Otto G.. (2009). Real-time DNA sequencing from single polymerase molecules. Sci. (New York N.Y.) 323 (5910), 133–138. doi: 10.1126/science.1162986 19023044

[B23] Flusberg B. A. Webster D. R. Lee J. H. Travers K. J. Olivares E. C. Clark T. A.. (2010). Direct detection of DNA methylation during single-molecule, real-time sequencing. Nat. Methods 7 (6), 461–465. doi: 10.1038/nmeth.1459 20453866 PMC2879396

[B24] Fornelos N. Browning D. F. Butala M. (2016). The use and abuse of LexA by mobile genetic elements. Trends In Microbiol. 24 (5), 391–401. doi: 10.1016/j.tim.2016.02.009 26970840

[B25] Gold M. Hurwitz J. Anders M. (1963). The enzymatic methylation OF RNA and DNA, II. On the species specificity of the methylation enzymes. Proc. Natl. Acad. Sci. USA 50 (1), 164–169. doi: 10.1073/pnas.50.1.164 16578536 PMC300670

[B26] Gu W. Miller S. Chiu C. Y. (2019). Clinical metagenomic next-generation sequencing for pathogen detection. Annu. Rev. Pathol. 14, 319–338. doi: 10.1146/annurev-pathmechdis-012418-012751 30355154 PMC6345613

[B27] Guo J. Bolduc B. Zayed A. A. Varsani A. Dominguez-Huerta G. Delmont T. O.. (2021). VirSorter2: a multi-classifier, expert-guided approach to detect diverse DNA and RNA viruses. Microbiome 9 (1), 37. doi: 10.1186/s40168-020-00990-y 33522966 PMC7852108

[B28] Han S. Liu J. Li M. Zhang Y. Duan X. Zhang Y.. (2022). DNA methyltransferase regulates nitric oxide homeostasis and virulence in a chronically adapted pseudomonas aeruginosa strain. MSystems 7 (5), e0043422. doi: 10.1128/msystems.00434-22 36106744 PMC9600465

[B29] Huang W. Hamouche J. E. Wang G. Smith M. Yin C. Dhand A.. (2020). Integrated genome-wide analysis of an isogenic pair of Pseudomonas aeruginosa clinical isolates with differential antimicrobial resistance to Ceftolozane/Tazobactam, Ceftazidime/Avibactam, and Piperacillin/Tazobactam. Int. J. Mol. Sci. 21 (3), 1026. doi: 10.3390/ijms21031026 32033143 PMC7037351

[B30] Hung C. L. Lin Y. S. Lin C. Y. Chung Y. C. Chung Y. F. (2015). CUDA ClustalW: an efficient parallel algorithm for progressive multiple sequence alignment on Multi-GPUs. Comput. Biol. Chem. 58, 62–68. doi: 10.1016/j.compbiolchem.2015.05.004 26052076

[B31] Juhas M. van der Meer J. R. Gaillard M. Harding R. M. Hood D. W. Crook D. W. (2009). Genomic islands: tools of bacterial horizontal gene transfer and evolution. FEMS Microbiol. Rev. 33 (2), 376–393. doi: 10.1111/j.1574-6976.2008.00136.x 19178566 PMC2704930

[B32] Kamruzzaman M. Wu A. Y. Iredell J. R. (2021). Biological functions of type II toxin-antitoxin systems in bacteria. Microorganisms 9 (6), 1276. doi: 10.3390/microorganisms9061276 34208120 PMC8230891

[B33] Kreuzer K. N. (2013). DNA damage responses in prokaryotes: regulating gene expression, modulating growth patterns, and manipulating replication forks. Cold Spring Harbor Perspect. In Biol. 5 (11), a012674. doi: 10.1101/cshperspect.a012674 PMC3809575 24097899

[B34] Li J. Li J.-W. Feng Z. Wang J. An H. Liu Y.. (2016). Epigenetic switch driven by DNA inversions dictates phase variation in Streptococcus pneumoniae. PloS Pathog. 12 (7), e1005762. doi: 10.1371/journal.ppat.1005762 27427949 PMC4948785

[B35] Li G. Shen M. Le S. Tan Y. Li M. Zhao X.. (2016). Genomic analyses of multidrug resistant Pseudomonas aeruginosa PA1 resequenced by single-molecule real-time sequencing. Biosci. Rep. 36 (6), e00418. doi: 10.1042/bsr20160282 27765811 PMC5293553

[B36] Lu S. Le S. Li G. Shen M. Tan Y. Zhao X.. (2015). Complete genome sequence of Pseudomonas aeruginosa PA1, isolated from a patient with a respiratory tract infection. Genome Announc. 3 (6), e01453–15. doi: 10.1128/genomeA.01453-15 PMC4675953 26659688

[B37] Nakano K. Shiroma A. Shimoji M. Tamotsu H. Ashimine N. Ohki S.. (2017). Advantages of genome sequencing by long-read sequencer using SMRT technology in medical area. Hum. Cell 30 (3), 149–161. doi: 10.1007/s13577-017-0168-8 28364362 PMC5486853

[B38] Oliveira P. H. Fang G. (2021). Conserved DNA methyltransferases: a window into fundamental mechanisms of epigenetic regulation in bacteria. Trends Microbiol. 29 (1), 28–40. doi: 10.1016/j.tim.2020.04.007 32417228 PMC7666040

[B39] Parkins M. D. Somayaji R. Waters V. J. (2018). Epidemiology, biology, and impact of clonal Pseudomonas aeruginosa infections in cystic fibrosis. Clin. Microbiol. Rev. 31 (4), e00019–18. doi: 10.1128/CMR.00019-18 30158299 PMC6148191

[B40] Pham T. H. Webb J. S. Rehm B. H. (2004). The role of polyhydroxyalkanoate biosynthesis by Pseudomonas aeruginosa in rhamnolipid and alginate production as well as stress tolerance and biofilm formation. Microbiol. (Reading England) 150 (Pt 10), 3405–3413. doi: 10.1099/mic.0.27357-0 15470118

[B41] Pleška M. Qian L. Okura R. Bergmiller T. Wakamoto Y. Kussell E.. (2016). Bacterial autoimmunity due to a restriction-modification system. Curr. Biol.: CB 26 (3), 404–409. doi: 10.1016/j.cub.2015.12.041 26804559

[B42] Quinaud M. Chabert J. Faudry E. Neumann E. Lemaire D. Pastor A.. (2005). The PscE-PscF-PscG complex controls type III secretion needle biogenesis in Pseudomonas aeruginosa. J. Biol. Chem. 280 (43), 36293–36300. doi: 10.1074/jbc.M508089200 16115870

[B43] Roberts R. J. Vincze T. Posfai J. Macelis D. (2022). REBASE: a database for DNA restriction and modification: enzymes, genes and genomes. Nucleic Acids Res. Null. 51 (D1), D629–D630. doi: 10.1093/nar/gkac975 PMC9825431 36318248

[B44] Schadt E. E. Turner S. Kasarskis A. (2010). A window into third-generation sequencing. Hum. Mol. Genet. 19 (R2), R227–R240. doi: 10.1093/hmg/ddq416 20858600

[B45] Som A. Fuellen G. (2009). The effect of heterotachy in multigene analysis using the neighbor joining method. Mol. Phylogenet. Evol. 52 (3), 846–851. doi: 10.1016/j.ympev.2009.05.025 19482090

[B46] Sonnhammer E. L. von Heijne G. Krogh A. (1998). “A hidden Markov model for predicting transmembrane helices in protein sequences,” in Proceedings. International Conference on Intelligent Systems for Molecular Biology 6, 175–182. 9783223

[B47] Stover C. K. Pham X. Q. Erwin A. L. Mizoguchi S. D. Warrener P. Hickey M. J.. (2000). Complete genome sequence of Pseudomonas aeruginosa PAO1, an opportunistic pathogen. Nature 406 (6799), 959–964. doi: 10.1038/35023079 10984043

[B48] Taguchi F. Ichinose Y. (2011). Role of type IV pili in virulence of Pseudomonas syringae pv. tabaci 6605: correlation of motility, multidrug resistance, and HR-inducing activity on a nonhost plant. Mol. Plant-Microbe Interactions: MPMI 24 (9), 1001–1011. doi: 10.1094/mpmi-02-11-0026 21615203

[B49] Tamura K. Stecher G. Kumar S. (2021). MEGA11: molecular evolutionary genetics analysis version 11. Mol. Biol. Evol. 38 (7), 3022–3027. doi: 10.1093/molbev/msab120 33892491 PMC8233496

[B50] Teufel F. Armenteros Almagro J. J. Johansen A. R. Gíslason M. H. Pihl SI. Tsirigos K. D.. (2022). SignalP 6.0 predicts all five types of signal peptides using protein language models. Nat. Biotechnol. 40 (7), 1023–1025. doi: 10.1038/s41587-021-01156-3 34980915 PMC9287161

[B51] Veetilvalappil V. V. Manuel A. Aranjani J. M. Tawale R. Koteshwara A. (2022). Pathogenic arsenal of Pseudomonas aeruginosa: an update on virulence factors. Future Microbiol. 17, 465–481. doi: 10.2217/fmb-2021-0158 35289684

[B52] Vernikos G. S. Parkhill J. (2006). Interpolated variable order motifs for identification of horizontally acquired DNA: revisiting the Salmonella pathogenicity islands. Bioinf. (Oxford England) 22 (18), 2196–2203. doi: 10.1093/bioinformatics/btl369 16837528

[B53] Winsor G. L. Griffiths E. J. Lo R. Dhillon B. K. Shay J. A. Brinkman F. S. (2016). Enhanced annotations and features for comparing thousands of Pseudomonas genomes in the Pseudomonas genome database. Nucleic Acids Res. 44 (D1), D646–D653. doi: 10.1093/nar/gkv1227 26578582 PMC4702867

[B54] Yang B. Wang Y. Qian P. Y. (2016). Sensitivity and correlation of hypervariable regions in 16S rRNA genes in phylogenetic analysis. BMC Bioinf. 17, 135. doi: 10.1186/s12859-016-0992-y PMC4802574 27000765

[B55] Yang X. Li Y. Zang J. Li Y. Bie P. Lu Y.. (2016). Analysis of pan-genome to identify the core genes and essential genes of Brucella spp. Mol. Genet. Genomics: MGG 291 (2), 905–912. doi: 10.1007/s00438-015-1154-z 26724943

[B56] Yang Y. Scott SA. (2017). DNA methylation profiling using long-read single molecule real-time bisulfite sequencing (SMRT-BS). Methods Mol. Biol. (Clifton N.J.) 1654, 125–134. doi: 10.1007/978-1-4939-7231-9_8 28986786

[B57] Zautner A. E. Goldschmidt A. M. Thürmer A. Schuldes J. Bader O. Lugert R.. (2015). SMRT sequencing of the Campylobacter coli BfR-CA-9557 genome sequence reveals unique methylation motifs. BMC Genomics 16, 1088. doi: 10.1186/s12864-015-2317-3 26689587 PMC4687069

[B58] Zhao X. L. Chen Z. G. Yang T. C. Jiang M. Wang J. Cheng Z. X.. (2021). Glutamine promotes antibiotic uptake to kill multidrug-resistant uropathogenic bacteria. Sci. Trans. Med. 13 (625), eabj0716. doi: 10.1126/scitranslmed.abj0716 34936385

