# Supplementary figures and images for: Comparative genomics and DNA methylation analysis of Pseudomonas aeruginosa clinical isolate PA3 by single-molecule real-time sequencing reveals new targets for antimicrobials
Source: Front Cell Infect Microbiol. 2023 Aug 18;13:1180194. doi: 10.3389/fcimb.2023.1180194 (PMC10471985; doi:10.3389/fcimb.2023.1180194)

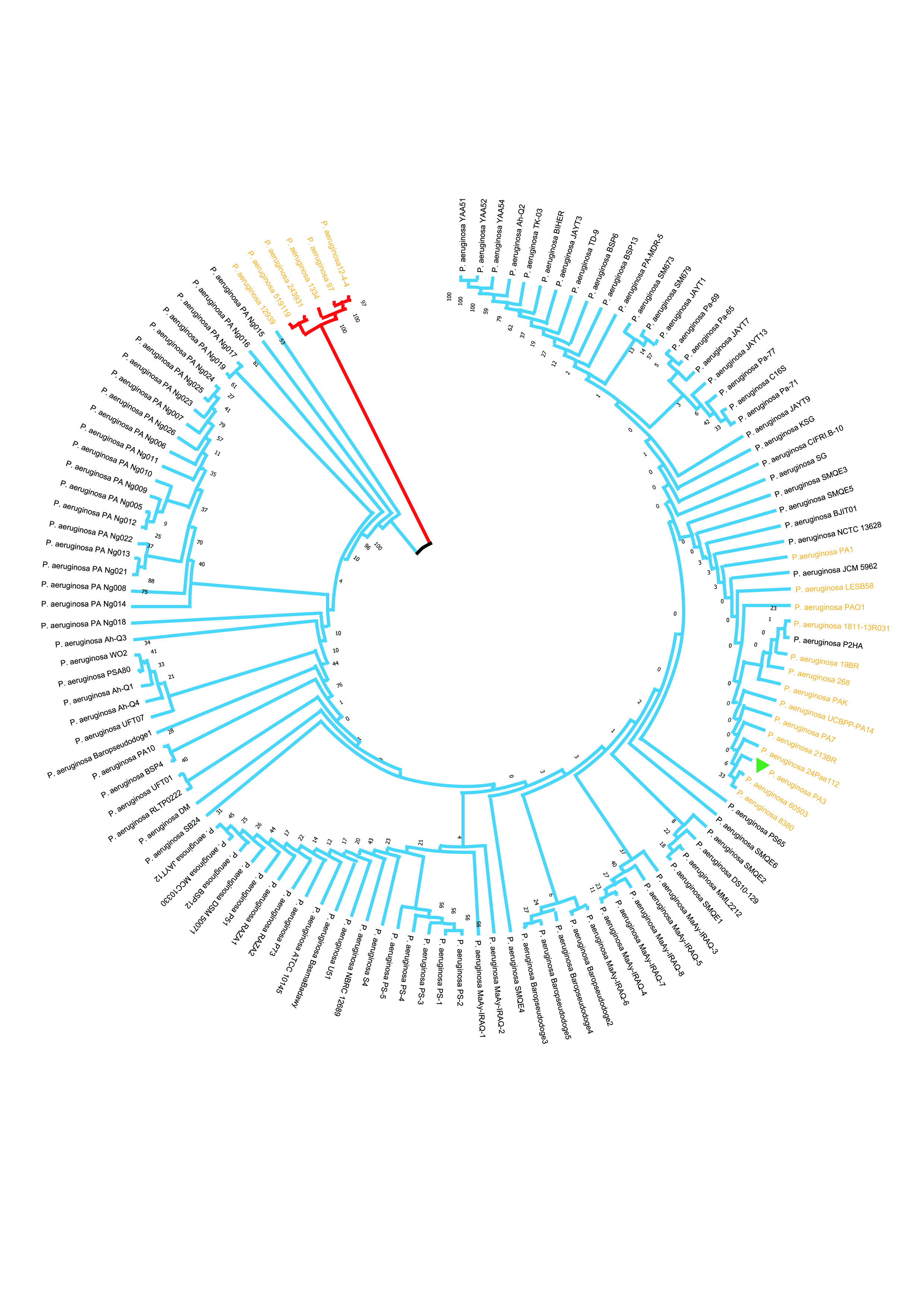

Supplement: Supplementary Figure 1 — The original phylogenetic tree of P. aeruginosa PA3. [file Image_1.tif]

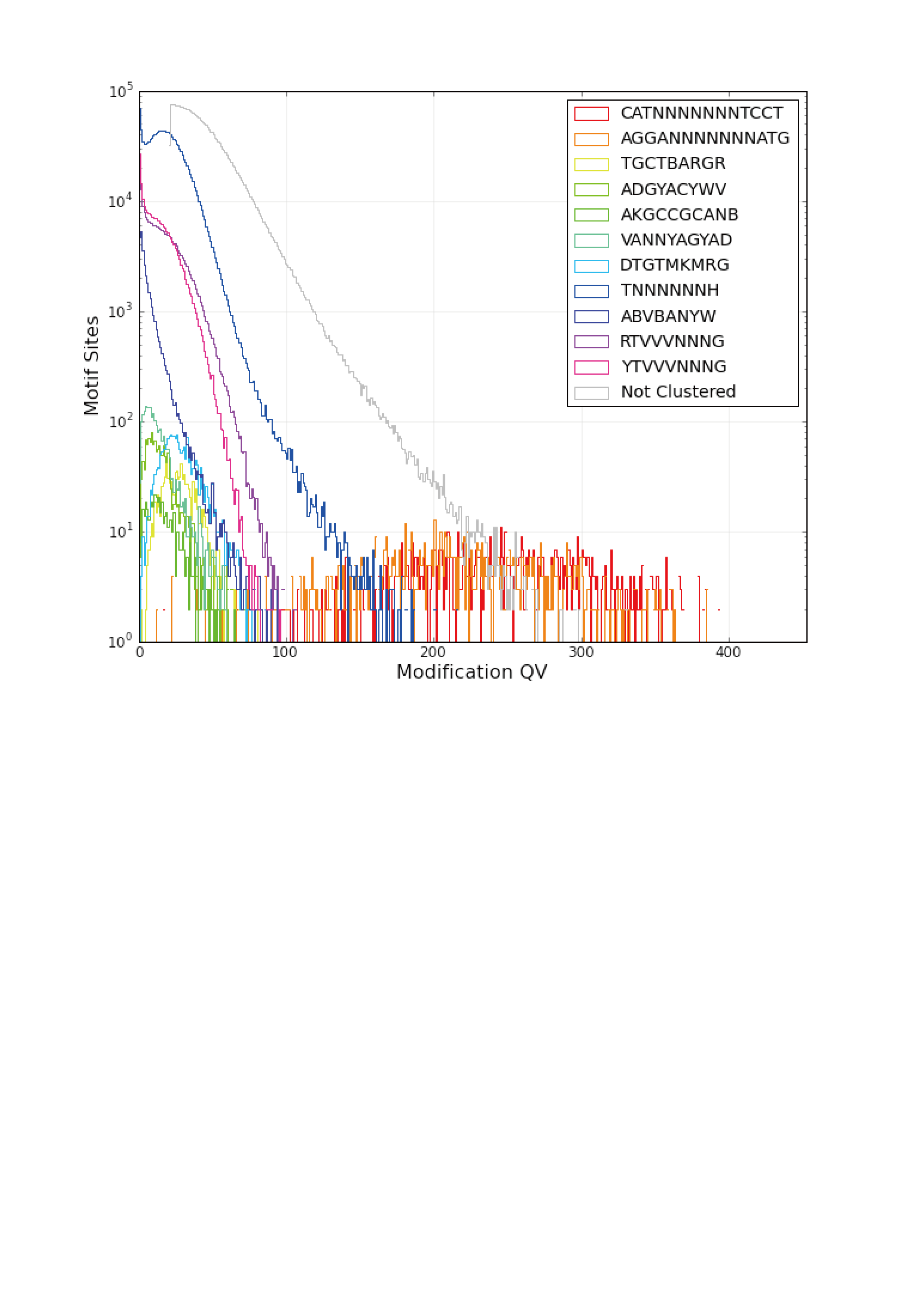

Supplement: Supplementary Figure 2 — The quality value (QV) and motif base count change of P. aeruginosa PA3. [file Image_2.tif]
