# Supplementary material for: Comparative genomics and DNA methylation analysis of Pseudomonas aeruginosa clinical isolate PA3 by single-molecule real-time sequencing reveals new targets for antimicrobials
Source: Front Cell Infect Microbiol. 2023 Aug 18;13:1180194. doi: 10.3389/fcimb.2023.1180194 (PMC10471985; doi:10.3389/fcimb.2023.1180194)
Supplement: Supplementary Table 8 — Drug sensitive tests of wide type PA3. [file Table_8.doc]

**Drug sensitive tests of wide type *P. aeruginosa* PA3**

| **Antibiotic** | **PA3** |
| --- | --- |
| Gentamicin | 22 |
| Pailaxilinna | 29 |
| Netilmicin | 22 |
| Ceftazidime Pentahydrate | 26 |
| Aztreonam | 26 |
| Meropenem | 24 |
| Polymyxin B | 17 |
| Amikacin | 26 |
| Ciprofloxacin | 25 |
| Tobramycin. | 25 |
| Cefepime | 28 |
| Imine Penem | 17 |
| Piperacillin/Tazobactam | 30 |
| Cefoperazone/Sulbactam | 24 |

Drug sensitive tests were performed by the Kirby Bauer disk diffusion method.
